# Quantification and Imaging of Nanoscale Contact with Förster Resonance Energy Transfer

**DOI:** 10.1021/acsami.1c04226

**Published:** 2021-04-15

**Authors:** Mónica G. Simões, Georg Urstöger, Robert Schennach, Ulrich Hirn

**Affiliations:** † Institute of Bioproducts and Paper Technology, Inffeldgasse 23, 8010 Graz, Austria; ‡ CD Laboratory for Fiber Swelling and Paper Performance, Inffeldgasse 23, 8010 Graz, Austria; § Institute of Solid-State Physics, Graz University of Technology, Petersgasse 16, 8010 Graz, Austria

**Keywords:** nanoscale contact, adhesion, contact mechanics, Förster resonance energy transfer, polymer films, FRET spectroscopy, FRET microscopy

## Abstract

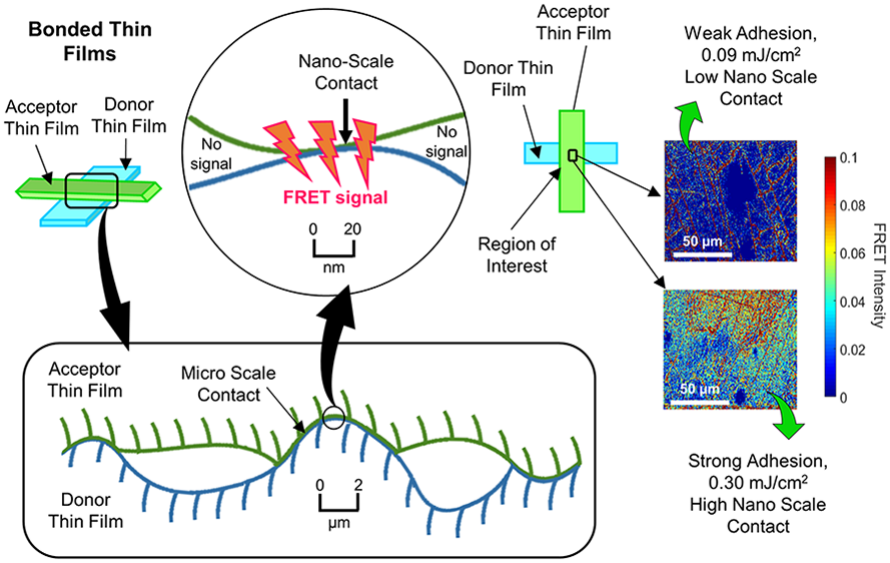

Adhesion is caused by molecular interactions that only take place if the surfaces are in nanoscale contact (NSC); i.e., the distance between the surfaces is in the range of 0.1–0.4 nm. However, there are several difficulties measuring the NSC between surfaces, mainly because regions that appear to be in full contact at low magnification may show no NSC when observed at higher magnifications. Thus, the measurement area of NSC is very small with imaging techniques, and an experimental technique to evaluate NSC for large contact areas has not been available thus far. Here, we are proposing Förster resonance energy transfer (FRET) spectroscopy/microscopy for this purpose. We demonstrate that NSC in a distance range of 1–10 nm can be evaluated. Our experiments reveal that, for thin films pressed under different loads, NSC increases with the applied pressure, resulting in a higher FRET signal and a corresponding increase in adhesion force/energy when separating the films. Furthermore, we show that local variations in molecular contact can be visualized with FRET microscopy. Thus, we are introducing a spectroscopic technique for quantification (FRET spectroscopy) and imaging (FRET microscopy) of NSC between surfaces, demonstrated here for the application of surface adhesion. This could be of interest for all fields where adhesion or nanoscale surface contact are playing a role, for example, soft matter, biological materials, and polymers, but also engineering applications, like tribology, adhesives, and sealants.

## Introduction

Adhesion between solid materials is crucial in several fields of biology, science, and technology, such as cellular adhesion and contact mechanics.^1−4^ It is caused by intermolecular forces, like hydrogen bonding and van der Waals forces.^5^ These mechanisms are taking place up to a distance of 0.1–0.4 nm^6,7^ and, thus, require nanoscale contact (NSC) between the adhering surfaces.^8,9^ As a result, an increase in nanoscale contact area (NSCA) leads to an increase in adhesion between surfaces.^10−12^

**Figure 1:**
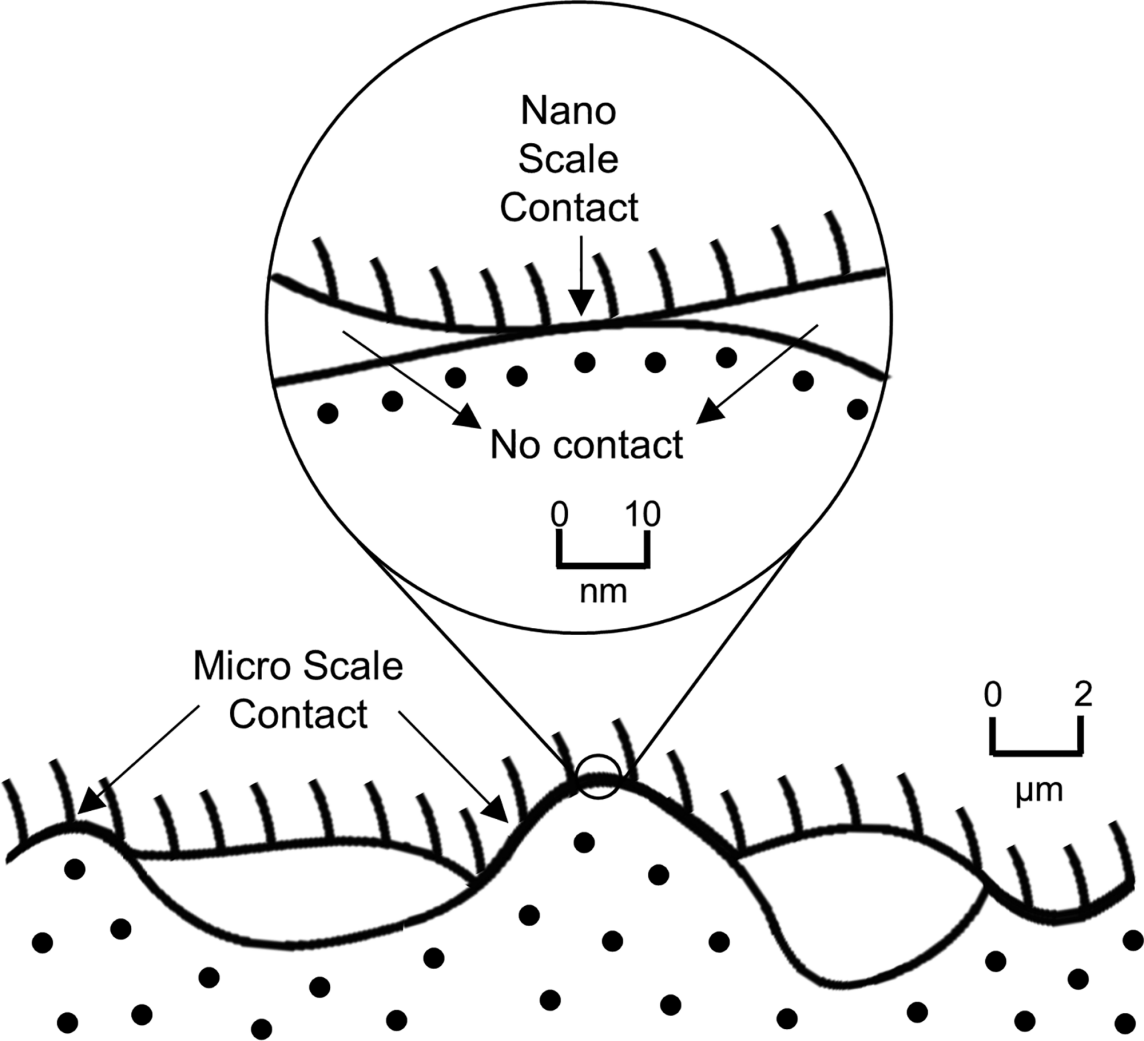
Two surfaces in physical contact observed at micro- and nanometer scales. The contact area decreases with increasing magnification.

However, it is challenging to quantify the NSCA. Surfaces in close contact observed under lower resolution frequently reveal gaps when inspected at the atomic length scale (Figure 1).^13−15^ Thus, the NSCA is usually lower (at maximum equal) than the contact observed at lower resolution.^16−18^ In conclusion, direct observation of NSCA is only possible with imaging techniques having a resolution in the length scale of the interaction forces, i.e., around 1 nm. Nevertheless, optical microscopy is sometimes used to estimate the contact between materials with low roughness.^16,19^ Also imaging of the contact area with scanning electron microscopy (SEM) resolution^17^ can only reveal microscale contact. Transmission electron microscopy (TEM) is able to image surface contact on the relevant length scale.^20−23^ However, high-resolution TEM is only suited for semi-quantitative inspection because the imaging area is below 1 μm^2^, which makes it very hard to conduct a statistically meaningful quantitative analysis. Also, the sample preparation for TEM is delicate and laborious. In conclusion, direct imaging techniques with low magnification are systematically overestimating the NSCA, and high-resolution imaging techniques can only provide qualitative indications as a result of the extremely small imaging area.

Other groups use force modulation with depth-sensing nanoindentation, by mapping and comparing the surfaces before and after being in contact to estimate the NSCA.^23−25^ However, this approach presents some disadvantages, e.g., the reduced sample size, low resolution, and possible recovery of the initial morphology/roughness of the material after it is no longer in contact. A key approach is contact mechanic simulations. Contact mechanic theories are used to study the NSCA dependence upon the scale of observation.^20,24,26−28^ The influence of roughness on NSC, friction, and adhesion between elastic bodies is also modeled by multiscale molecular dynamics.^8,29^ Modeling-based approaches are currently the best available techniques to estimate NSCA. To the best of our knowledge, no experimental technique is available to evaluate NSCA for an inspection area above the nanometer scale. In this work, we are proposing such a method, applicable up to the millimeter-scale sample area. We are employing Förster resonance energy transfer (FRET) spectroscopy for (a) quantification of NSC and (b) FRET microscopy for two-dimensional (2D) imaging of local variations in NSC. Such an experimental method to evaluate NSC could be useful to study adhesion between surfaces for soft matter and biological materials but also engineering applications, like tribology, adhesives, and polymers.

FRET is a technique capable of measuring the nanometric distance (0–20 nm) between surfaces in close contact.^30−32^ For that, each surface is labeled with a fluorescence dye, donor or acceptor. FRET uses the non-radiative energy transferred between the donor and acceptor molecules to study their exact nanometric distance. The distance range of FRET depends upon the Förster radius (*R*_0_) of the selected dye system. If the molecules are close enough to each other, i.e., below the critical distance of 2*R*_0_, a FRET signal can be detected.

FRET is commonly used in biological/biomedical applications to confirm NSC between molecules in studies related to, e.g., protein/cellular adhesion.^33−36^ FRET microscopy and spectroscopy have also been used to study interdiffusion between polymeric materials.^30,37−39^

**Figure 2:**
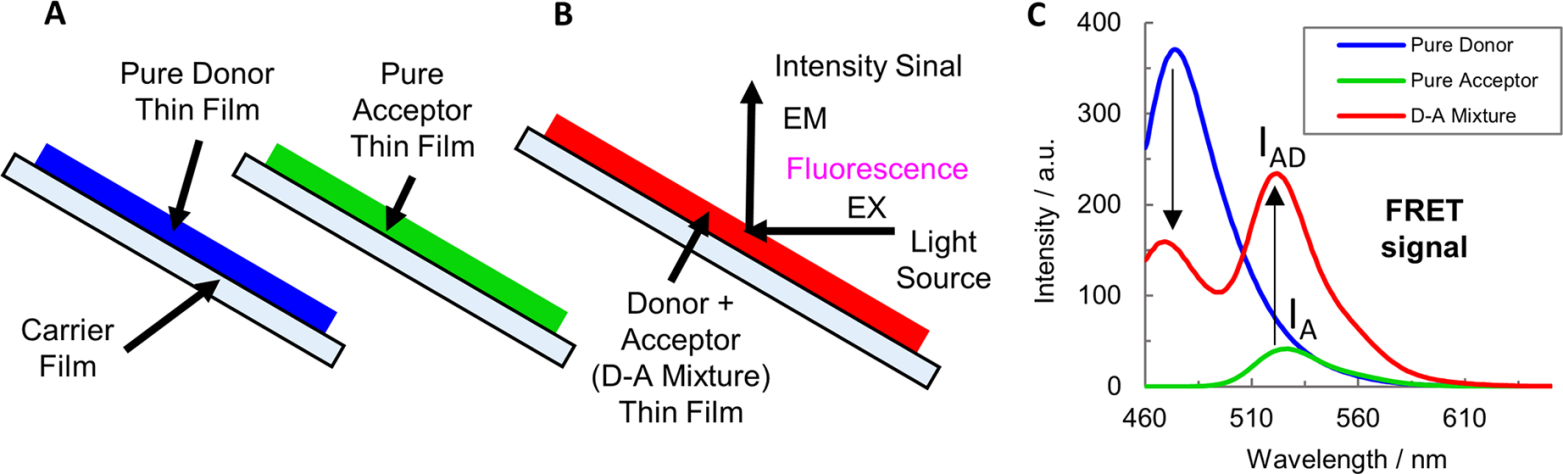
FRET signal: (A) donor and acceptor individual thin films, (B) donor–acceptor mixture thin film, and (C) pure donor, acceptor, and donor–acceptor mixture fluorescence spectra.

To apply FRET, donor and acceptor molecules must present different yet overlapping emission and excitation fluorescence spectra, respectively. By exciting them at the same excitation wavelength, the energy transfer can be observed.^40^Figure 2 shows a basic experiment to demonstrate FRET between a pair of one donor and one acceptor dye uniformly distributed in pHema thin films. First, their emission spectra are collected for the individual dyes (a) and then for a mixture of the dyes (b). Energy transfer between the dyes, i.e., a FRET signal, is identified when, in comparison to the spectra of the pure dyes, in the mixture, the intensity of the donor dye is dropping (left downward arrow) and the intensity of the acceptor dye is increasing (right arrow up from *I*_A_ to *I*_AD_; see Figure 2C). Please note that the energy transfer between the dyes (in this case, FTSC and DCCH) can only take place between donor and acceptor molecules closer than 2*R*_0_ < 10.2 nm, which is obviously the case in the mixture (Figure 2B).

**Figure 3:**
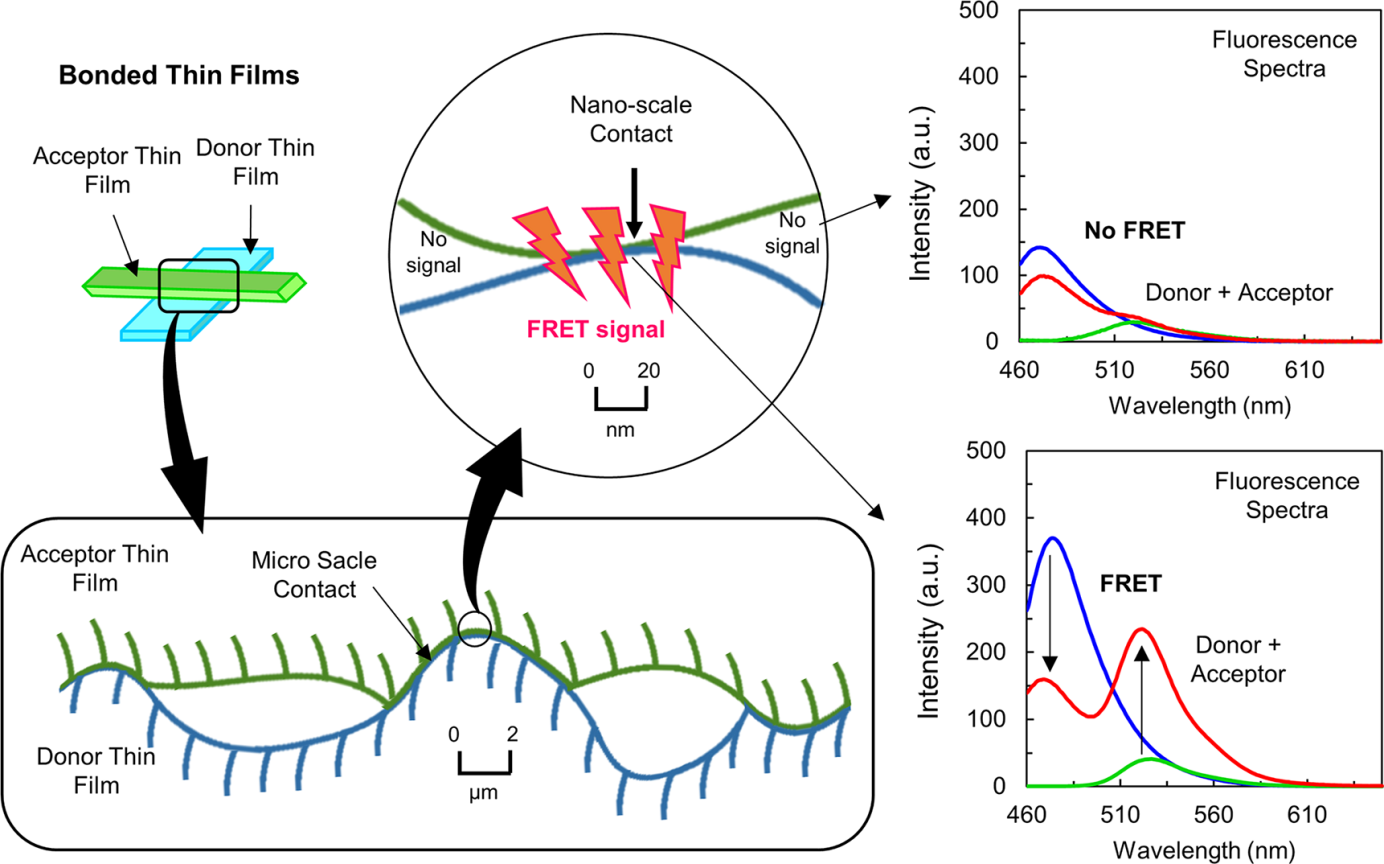
Donor- and acceptor-labeled thin films in close physical contact observed at micro- and nanometer scales. NSC decreases with increasing magnification. For NSC, a FRET signal can be observed between donor and acceptor surfaces and the signal does not occur for surfaces with a distance larger than 10 nm.

In this work, we are quantifying the degree of NSC between thin films bonded together. Therefore, one film is labeled with a FRET donor dye, and another film is labeled with a FRET acceptor dye (Figure 3). The degree of NSC between bonded films is evaluated by calculating the FRET energy transfer efficiency (FRET efficiency)^31^ on the interface between two films, as seen in Figure 3. Then, the measured degree of NSC is correlated to the adhesion between the films, which have been bonded together with ascending pressure, thus leading to an increased degree of NSC and, as a consequence, to an increased adhesion between the films. By demonstrating the correlation between the FRET signal and the measured separation energy between the thin films, we are validating our approach to quantify NSC using FRET spectroscopy.

Finally, we are demonstrating that FRET microscopy can be used to image local variations in NSC between the bonded films. For FRET microscopy, the microscope is equipped with a set of fluorescence filters specific to the FRET dyes used. Images acquired with the different filter sets can be analyzed to obtain a local FRET intensity in every image pixel using FRET algorithms, as provided, e.g., by Gordon et al. and Xia et al.^41,42^ For each image pixel, we obtain a dimensionless value (NFRET) indicating the local degree of NSC between the interfaces,^30,43^ thus generating a map imaging the local variation of NSC over the sample area.

## Results and Discussion

**Figure 4:**
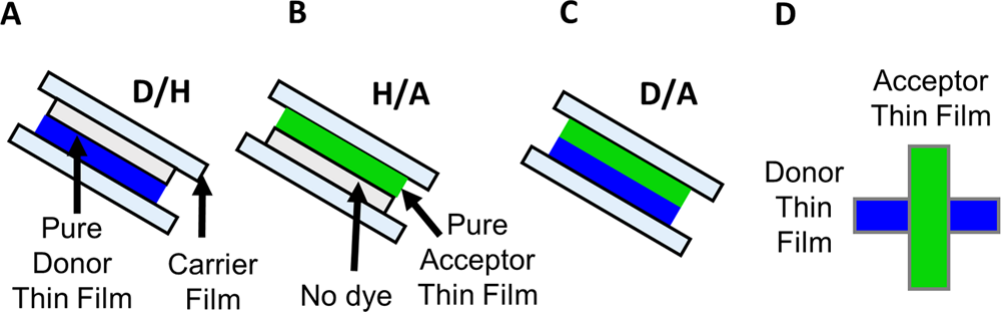
Thin films bonded by pressing with 1.5–150 bar: (A) donor/pHema (D/H), (B) pHema/acceptor (H/A), and (C) donor/acceptor (D/A) bonded thin films and (D) cross-bonded D/A thin films.

For the FRET system, 7-(diethylamino)coumarin-3-carbohydrazide (DCCH, donor) and fluorescein-5-thiosemicarbazide (FTSC, acceptor) were selected as the fluorescence dyes.^44^ At the dye concentration used in the experiments (see the Methods section), the DCCH/FTSC system presents a FRET distance range (2*R*_0_) of 0–10.2 nm (*R*_0_ = 5.1 nm), which allowed us to study NSC with this FRET pair. All thin films consisted of pHema and were produced by doctor blading. The dyes were mixed into pHema (Figures 2 and 4) at an equivalent molar concentration of 1.5 mM. The thickness (1.5 ± 0.1 μm) and low roughness (0.04 ± 0.01 μm) of the thin films show a uniform distribution of the dyes, which is of extreme importance for a correct FRET result.

**Figure 5:**
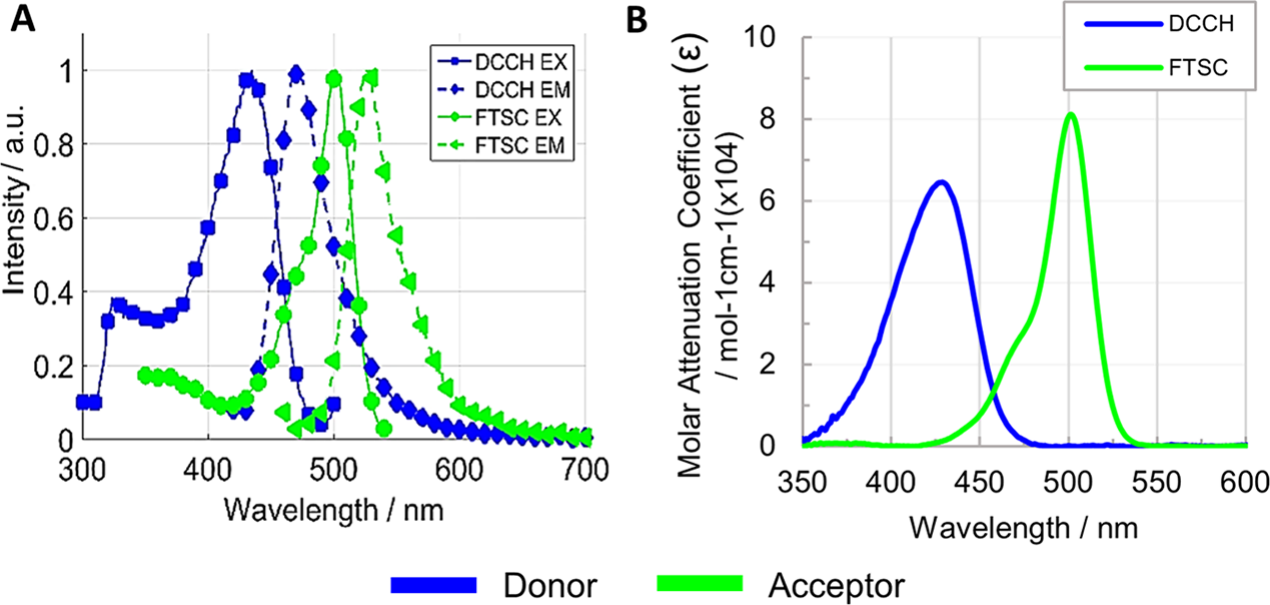
Donor (DCCH) and acceptor (FTSC) thin film fluorescence properties as a FRET pair: (A) excitation and emission florescence spectra and (B) molar attenuation coefficient spectra.

The fluorescence spectra (Figure 5A) of the pure donor and acceptor thin films (Figure 2A) show the DCCH emission and FTSC excitation spectra overlapping area, which is necessary for FRET.^31,44^ The spectra were measured on thin films bonded in the configuration shown in panels A and B of Figure 4.

The spectra (Figure 5B) of the molar attenuation coefficients (ε_D_ and ε_A_) were calculated from the absorbance spectra of pure donor and pure acceptor thin films (eq 4). ε_D_ and ε_A_ spectra (Figure 5B) show how well the dyes absorb the light and the regions where both dyes can be analyzed at the same excitation wavelength. We chose to collect the fluorescence spectra and FRET measurements at 440 nm excitation, finding a ratio of ε_A_/ε_D_ = 0.07.

First, individual thin films of the pure donor, pure acceptor (Figure 2A), and a donor–acceptor (D–A) mixture (Figure 2B) are investigated as a positive control. In the D–A thin film both donor and acceptor dye are mixed, leading to short distances between the molecules in the thin-film polymeric matrix. Therefore, the D–A mixture thin film represents the maximum value of FRET efficiency that the system can reach, at this dye concentration. Figure 2C exhibits the fluorescence spectra where a strong FRET signal can be observed (drop in the donor intensity and an increase of the acceptor signal; see arrows). The FRET efficiency in acceptor sensitization measured for this system was FRETeff = 30.5%.

To validate FRET as a measurement for NSC between surfaces, we bonded dyed polymer thin films by pressing the surfaces together with a pressure from 1.5 to 150 bar.

**Figure 6:**
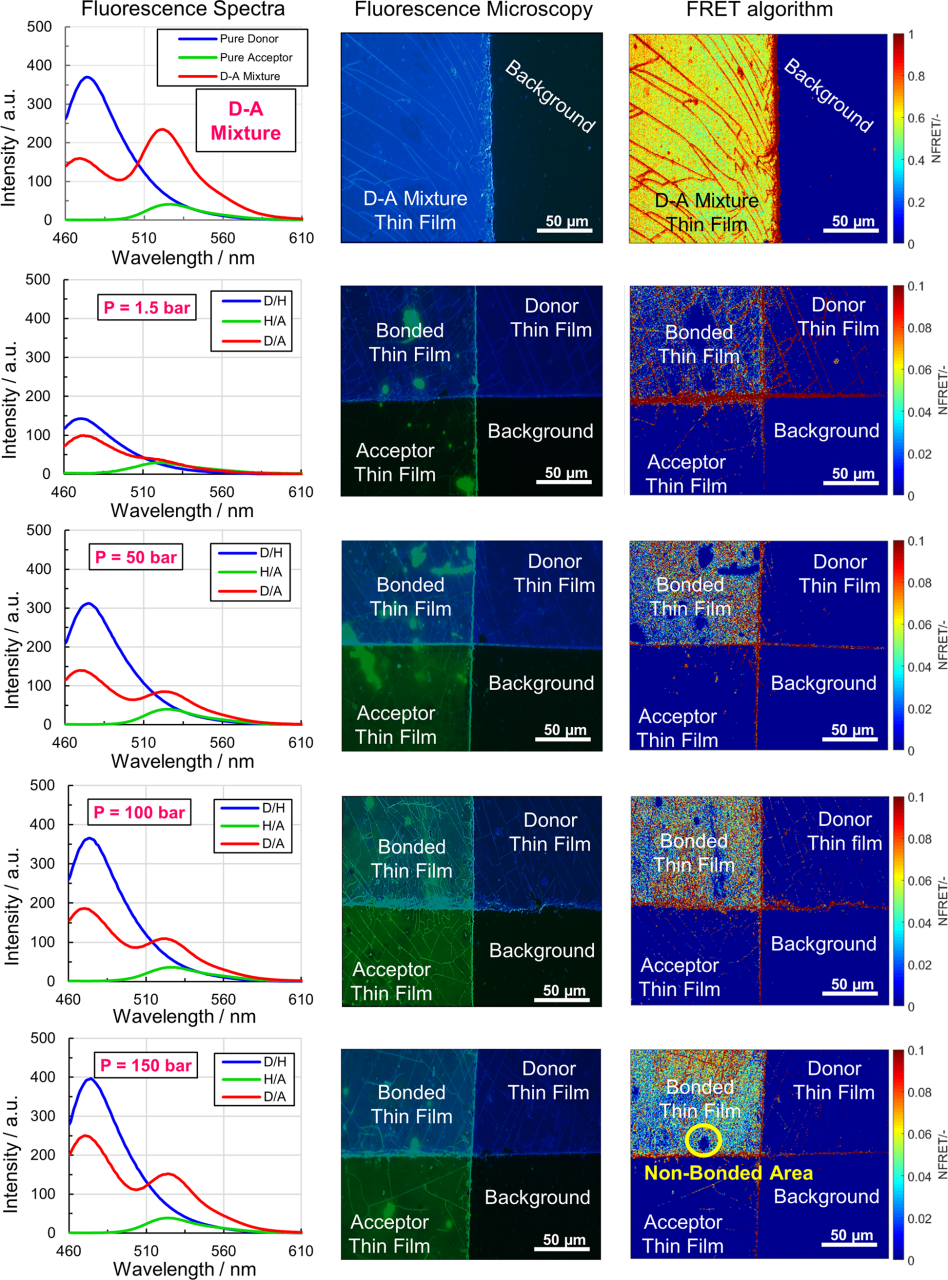
Maximum FRET signal in the positive control (top row), furthermore bonded thin films with increasing bonding pressure from 1.5 to 150 bar (other rows downward). Fluorescence spectra (left column). False color images of the thin films taken with fluorescence microscopy, with 50× magnification (middle column). NFRET intensity maps (right column) indicating the local variation in NSC between the surfaces; non-bonded regions (right column, bottom row) can also be identified.

The films were prepared by bonding pure donor (D), pure acceptor (A), and films with no dye (H) (panels A and B of Figure 4). The arrangement of bonded thin films (panels C and D of Figure 4) was the same for all measurements, with the donor in the back position and acceptor in the front position. The left column of Figure 6 depicts the fluorescence spectra of the bonded thin films bonded with the lowest (1.5 bar) and highest (150 bar) pressure, as given in panels A and B of Figure 4.

FRET efficiencies were calculated by the acceptor sensitization method^30,31^ (Table 1), which only relies on the acceptor performance (eq 3). The positive control (D–A mixture thin film) revealed a FRET efficiency of ∼30%. When the pressure to bond the thin films is increased, the FRET signals (left column of Figure 6) and their corresponding FRET efficiencies (Table 1) also increase accordingly. For the minimum bonding pressure of 1.5 bar, the measured FRET efficiency was 1.1%, and for the maximum bonding pressure of 150 bar, the FRET efficiency was 10%.

**Table 1:** FRET Efficiency, NFRET, Maximum Tensile Force, and Separation Energy Per Unit Area of the Individual and Bonded Thin Films Prepared under Different Pressures^a^

sample	FRET efficiency (%)	NFRET (2 × 10^2^)	maximum tensile force (N)	separation energy per unit area (mJ/cm^2^)
D–A mixture	30.5 ± 1.1	125.0 ± 0.6		
D/A, 1.5 bar	1.2 ± 0.4	7.2 ± 0.04	18.7 ± 1.2	0.09 ± 0.02
D/A, 50 bar	3.6 ± 0.7	8.1 ± 0.01	25.1 ± 1.1	0.17 ± 0.01
D/A, 100 bar	7.1 ± 1.1	8.7 ± 0.11	32.6 ± 1.6	0.22 ± 0.01
D/A, 150 bar	9.7 ± 1.2	9.6 ± 0.06	41.8 ± 2.3	0.30 ± 0.03

a FRET efficiency was determined by acceptor sensitization (at 440 nm, ε_A_/ε_D_ = 0.07; see Figure 5B). Values are the average ± 95% confidence interval (*n* = 3 for FRET efficiency; *n* = 6 for NFRET; and *n* = 10 for tensile force and separation energy).

**Figure 7:**
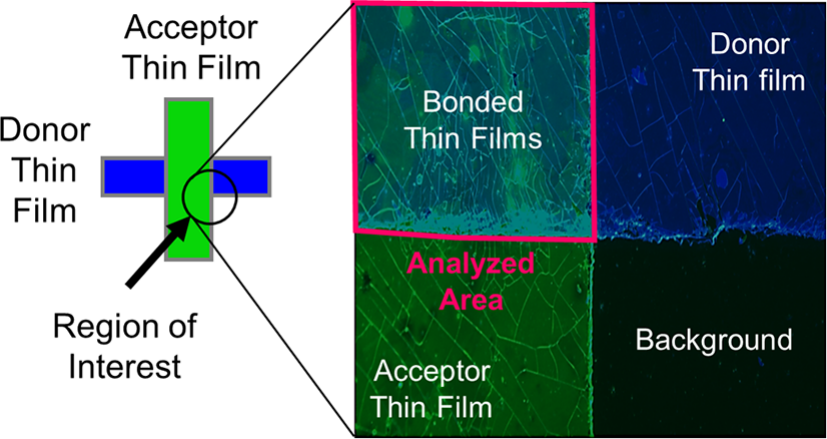
False color image of the bonded thin films taken with fluorescence microscopy, drawing the bonded area, region of interest, marked in magenta.

The thin films bonded at increasing pressure were also analyzed with FRET microscopy to demonstrate the capability to image local variations in NSC (middle and right columns of Figure 6) and validate FRET microscopy. For FRET microscopy, the efficiency of the energy transfer at the interface between the donor and acceptor thin films and, thus, the NSCA is measured by the NFRET value. For each image pixel, the local NFRET value indicates the degree of NSC in this image pixel. The donor–acceptor mixture thin film was again used as a reference, giving a maximum NFRET value of 125 (Table 1 and top row of Figure 6). For the remaining tests, the thin films were cross-bonded, and the images were captured in the region of interest (Figure 7). In the region of interest, only the top left part shows the bonded thin films; thus, only this part should show a NFRET signal. The other regions, showing donor film, acceptor film, and background, should not give a NFRET signal.

In the NFRET microscopy maps (right column of Figure 6), as expected, only the left top regions of the overlapping, bonded thin films exhibit a NFRET signal. Apart from some edge artifacts, the pure donor and acceptor films are showing no FRET signal, as expected. In the bonded regions, there is low NFRET intensity of the 1.5 bar thin films (second row of Figure 6), indicating very low NSC, and high intensity in the bonded area of the 150 bar thin films (bottom row of Figure 6), demonstrating high NSC between the donor and acceptor interfaces. Analyzing all bonded thin films (Figure 6), the same tendency observed before with the FRET spectroscopy experiments can be seen: when the pressure applied in the bonded thin films increases, the average NFRET values also increase accordingly (see the third column of Table 1). Both, FRET spectroscopy and FRET microscopy are thus able to correctly indicate the degree of NSC in the bonded polymer films.

Moreover, inspecting the FRET microscopy images, one can see that local variations in NSC, like, e.g., unbonded regions (right column, bottom row of Figure 6), can be identified. This indicates that FRET microscopy is indeed able to image NSC in the range of 1–10 nm for sample areas in the millimeter scale, bridging a scale difference in the order of 10^6^.

**Figure 8:**
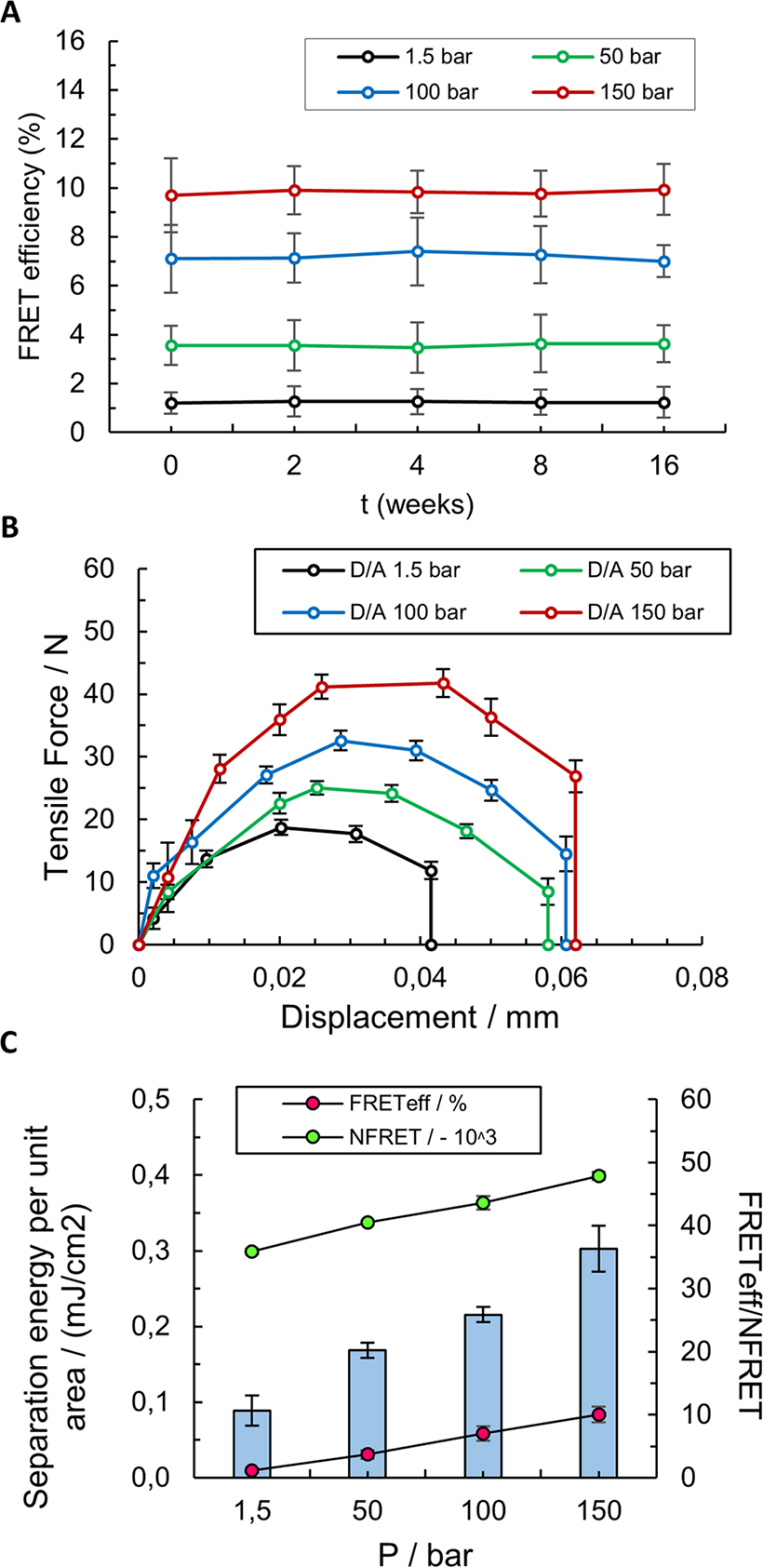
Thin films bonded by pressing with 1.5–150 bar, corresponding tensile force, FRET efficiency, and separation energy: (A) FRET efficiency of the D/A thin films bonded at different pressures over time, (B) separation force curves from bonded donor/acceptor thin films, and (C) FRET efficiency, NFRET, and separation energy per area of the D/A bonded thin films. The presented results refer to the mean average ± 95% confidence interval (*n* = 3).

Considering that donor and acceptor molecules are dissolved in the polymeric thin-film matrices, this could lead to migration of dye molecules into the opposing thin film, causing false FRET signals. Hence, the bonded thin films were analyzed by FRET spectroscopy over 16 weeks (Figure 8A). FRET efficiency has not significantly changed over time, which indicates no interdiffusion phenomena. Only NSC is responsible for the FRET signals.

Finally, the interrelation between NSC measured by FRET and surface adhesion was investigated. The bonded thin films were detached by *z*-directional tensile testing to measure the maximum adhesion force and separation energy of the films bonded with different pressures. The results show that maximum tensile force (Table 1) as well as separation energy are gradually increasing with increasing adhesion, i.e., separation force and separation energy (Figure 8B). The thin film separation energies per unit area were calculated by integrating the force–displacement curves of the tensile test and plotted in Figure 8C together with the FRET energy transfer efficiency between the bonded surfaces for FRET spectroscopy (FRET efficiency, red markers) and FRET microscopy (NFRET, green markers), as depicted in Figure 8B. With linearly increasing adhesion, the intensity of both FRET spectroscopy and FRET microscopy are also increasing linearly. The thin films are chemically and physically identical, and the differences in adhesion only descend from differences in NSC caused by increased pressing during the bonding. This proves that FRET spectroscopy and FRET microscopy are indeed a suitable approach to quantitatively investigate NSC between surfaces for millimeter-sized regions.

## Conclusion

We have presented a proof of concept that FRET methods can be used to quantify the degree of NSC between surfaces. Our system of FRET dyes, DCCH and FTSC, indicates surface contact in the range of 1–10 nm or closer. The FRET efficiency measured by acceptor sensitization in FRET spectroscopy is corresponding to an increase in NSC and adhesion between polymeric thin films. Measurements of NFRET intensity with FRET microscopy are confirming this relation. An implementation of FRET microscopy furthermore enables spatially resolved analysis of NSC. We believe that this novel concept to quantify and image NSC contact for large surface contact areas using optical methods, namely, FRET spectroscopy and FRET microscopy, provides a useful experimental approach to study different kinds of phenomena related to nanoscale surface contact, like adhesion, friction, interdiffusion, and contact mechanics, in general. This could be of interest for all fields where nanoscale surface contact is playing a role, for example, soft matter, biological materials, and polymers, but also engineering applications, like tribology, adhesives, and sealants.

## Methods

### Thin-Film Preparation

DCCH (SC-214392, Santa Cruz Biotechnology, Dallas, TX, U.S.A.), and FTSC (SC-211522, Santa Cruz Biotechnology, Dallas, TX, U.S.A.) were dissolved in tetrahydrofuran (3 mM). Donor–acceptor mixture solutions were prepared in a ratio of 1:1 (1.5 mM). A total of 100 μL of dye(s) solution was added to 500 μL of 10% (m/v) pHema (Mw 20 000 Da, CAS Registry Number 25249-16-5, Sigma-Aldrich, St. Louis, MO, U.S.A.) solution in an ethanol/Milli-Q water mixture 95:5 (v/v) and 5 μL of triethylamine, to ensure alkaline conditions.^44^ The polymeric solutions were doctor-bladed over polyvinyl chloride carrier films using a bar film applicator (3M BYK-Gardner GmbH, Geretsried, Germany) and left at room temperature for the evaporation of the solvents and consolidation of the films.

### Thin-Film Characterization

The thickness of the thin films was determined with a Bruker DekTak XT surface profiler. The scan length was set to 1000 μm over the time duration of 3 s with the hills and valleys scanning profile. The diamond stylus had a radius of 12.5 μm, and the employed force was 3 mg. The measured profile was then used to determine the thickness. Each layer thickness has been determined by averaging six measurements on three different spots on the thin films.

### Bonded Thin-Film Preparation

For FRET spectroscopy, the interface between the thin films was achieved by bonding 4 cm^2^ of pure donor (D), pure acceptor (A), and/or pHema thin films without any dye (H), as demonstrated in panels A–C of Figure 4. For FRET microscopy the contact was obtained by cross bonding 0.5 cm of pure donor and pure acceptor thin films, as shown in Figure 4D. For all samples, the thin films were pressed at 1.5, 50, 100, and 150 bar (hydraulic pressure PU30, V. Jessernigg & Urban, Graz, Austria) for 10 min at room temperature.

### FRET Spectroscopy

Spectra measurements of the individual and bonded thin films (Figures 2 and 4) were recorded using a spectra fluorophotometer RF-5301PC (Shimadzu, Kyoto, Japan), at an excitation wavelength of 440 nm in a 45°/45° configuration, as demonstrated in Figure 2B.

FRET signals were detected from the individual/bonded thin films and analyzed by the Förster theory.^31^ The dye system presents a FRET working range distance that corresponds to 2*R*_0_, where *R*_0_ is the Förster radius (nm). It that can be calculated via eq 1
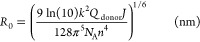
1where *N*_A_ is Avogadro’s constant (6.02 × 10^20^ mM^–1^), *k*^2^ is the orientation factor (^2^/_3_), *n* is the refractive index (1.5 for the thin films), *Q*_donor_ is the donor quantum yield (measured by the absolute method, *Q*_donor_ = 0.14 and *Q*_acceptor_ = 0.12), and *J* (nm^4^ M^–1^ cm^–1^) is the spectral overlap integral calculated with eq 2
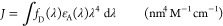
2where *f*_D_ is the donor emission spectrum normalized to unity (Figure 5A), ε_A_ is the attenuation coefficient of the acceptor (×10^4^ M^–1^ cm^–1^) (Figure 5B), and λ is the wavelength (cm^–1^).

FRET efficiency (%) was calculated by the acceptor sensitization method^1^ (eq 3). It is the ratio of the acceptor spectral intensity peak value in the presence (*I*_AD_) and absence (*I*_A_) of the donor (Figure 2C). To achieve appropriate FRET efficiency results, the direct luminescence of *I*_A_ is subtracted from *I*_AD_ and multiplied by the correct luminescence ratio of the acceptor and donor molar attenuation coefficients (ε_A_ and ε_D_) at the excitation wavelength used for the FRET experiments (Figure 5B).
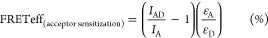
3The molar attenuation coefficients ε (×10^4^ M^–1^ cm^–1^) (Figure 5B) are determined from the absorbance by Beer–Lambert’s law (eq 4)
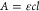
4where *A* is the absorbance defined as the negative decadic logarithm of the measured transmittance, *c* is the concentration of the dye in the polymeric matrix (mM), and *l* is the length of the light path, in this case, the thickness of the thin films (cm). Pure donor and pure acceptor thin film absorbance was measured with a Varian Cary ultraviolet–visible (UV–vis) spectrophotometer (Agilent Technologies, Santa Clara, CA, U.S.A.). To minimize the inner filter effect and deviations from Beer–Lambert’s law, the optical density of the transmission measurements never exceeded 0.5 optical density (OD).

### FRET Microscopy

Individual and bonded thin films were investigated using a wide field microscopy setup equipped with FRET filter sets (Table 2) customized to the dye excitation and emission spectra, operated with a 50 W tungsten halogen lamp. Images were taken with an optiMOS Scientific CMOS camera (QImaging, Canada) attached to the microscope via a C-mount interface.

**Table 2:** Fluorescence Microscopy Filter Sets Used to Study the Thin Films

filter set	excitation (nm)	dichroic mirror (nm)	emission (nm)
donor	436 ± 10	455 long pass	480 ± 20
acceptor	500 ± 10	515 long pass	520 long pass
FRET	436 ± 10	515 long pass	520 long pass

Both, the intensity of the lamp and the detector sensitivity show a dependency of the wavelength and were corrected by calculating correction factors from the lamp emission spectrum folded with the excitation filters, and the extinction coefficient and the detector sensitivity folded with the emission filters and the emission spectra.

Images were taken with an optiMOS Scientific CMOS camera (QImaging, Canada). All samples were investigated using a 50× magnification, and to minimize background noise, the microscope was operated in a dark space without ambient light.

The equations for the calculation of the FRET intensity are based on an algorithm developed by Gordon et al. (eqs 5–7).^41^ The method makes use of images recorded with the three different filter sets (Table 2), resulting in nine pictures for the individual thin films (pure donor, pure acceptor, and donor–acceptor mixture thin films) and three images for the bonded thin films (that contain in the same region/picture pure donor, pure acceptor, and bonded area; Figure 7).

For a detailed description of the algorithm, please refer to the original paper. In brief, this method calculates the FRET intensity corrected for all possible spectral bleed-through scenarios according to the following equations:
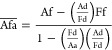
5
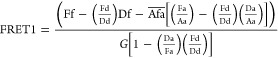
6
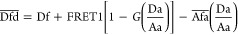
7The equations consist of variables with two letters. The first letter stands for the used filter set (Table 2), and the second letter stands for the investigated sample (d = donor only, a = acceptor only, and f = FRET area). For example, Af therefore stands for the FRET region (bonded area) investigated with the acceptor filter set. The variables represent the measured light intensities from the aforementioned microscope images recorded as 16-bit gray values. Afa refers to the acceptor signal that would have been if no donor were present and, therefore, no FRET occurred. Similarly, Dfd refers to the donor signal that would have been if no acceptor were present and, therefore, no FRET occurred. eqs 5–7 are used to calculate a FRET intensity normalized by the amount of donor and acceptor signals. To ensure a properly normalized and dimensionless quantity, Xia and Liu used the Gordon algorithm to calculate the improved property NFRET (eq 8),^42^ which we are using.
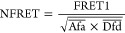
8The evaluation is performed pixelwise; i.e., for each pixel, an according NFRET value is calculated as the resulting NFRET intensity. *G* is an instrument and setup specific factor relating the loss of the donor signal to the increase of the acceptor signal. For thin films and the setup employed in this study, it amounted to 0.758.

To study NSC, a method was developed to select the appropriate area. The method consisted of manually drawing the bonded area (Figure 7, with regions of interest marked in magenta). Subsequently, we applied 5 pixel image erosion to remove the edge regions of the thin films, thus obtaining the eroded bonded area, which was then used for evaluation of FRET intensity. Removing the edge regions is necessary because regions of extreme NFRET intensity are appearing at the edges, which are a false-positive FRET signal.

### Thin-Film Separation Energy

The *z*-direction tensile tests were performed in a ZwickRoell Z010 multipurpose tester (Kennesaw, GA, U.S.A.) equipped with two steel bars, in which only the upper steel bar moves, driven by a linear motor. The *z*-direction tensile tests were performed in a Zwick Roell Z010 (Kennesaw, GA, U.S.A.) equipped with lower and upper steel bars, in which the upper steel bar moves up and down thanks to a linear motor. A double-sided adhesive tape is put on the upper and lower steel bars. After the sample is placed on the lower steel bar, the linear motor moves the upper steel bar down until it touches the sample. To guarantee good attachment of the sample at the steel bars, a defined compression force of 1.5 bar is applied. Then, the sample is pulled apart in the *z* direction until it fails between the two polymer thin films. The force *F* with respect to the separation distance *x* is recorded. The two main values for interpreting the tensile tests are the maximum tensile force and the separation energy. The separation energy is the integral of the force–distance curves.
